# Prosthetic rehabilitation of the geriatric oncologic rhinectomy patient utilizing a craniofacial implant‐retained nasal prosthesis

**DOI:** 10.1002/ccr3.2629

**Published:** 2019-12-26

**Authors:** Evan B. Rosen, Zain Uddin Ahmed, Joseph M. Huryn, Ian Ganly

**Affiliations:** ^1^ Dental Service Department of Surgery Memorial Sloan Kettering Cancer Center New York NY USA; ^2^ Miami Cancer Institute Miami FL USA; ^3^ Head and Neck Service Department of Surgery Memorial Sloan Kettering Cancer Center New York NY USA

**Keywords:** craniofacial implants, facial defect, nasal prosthesis, squamous cell carcinoma

## Abstract

This clinical report describes the expeditious treatment of a geriatric patient with squamous cell carcinoma of the nose treated with total rhinectomy, craniofacial implant placement, and a nasal prosthesis.

## Graphical abstract

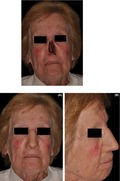

## 1 INTRODUCTION

Esthetic and functional rehabilitation of a geriatric patient requiring rhinectomy presents challenges for both the patient and the clinician. This clinical report describes the treatment sequence and management considerations for a geriatric patient with squamous cell carcinoma of the nose treated with total rhinectomy, craniofacial implant placement, and a nasal prosthesis.

Malignancies involving the nasal vestibule are rare and comprise 1% of all head and neck squamous cell carcinomas.^1^ Treatment for nasal squamous cell carcinoma may include ablative surgery with or without adjuvant therapy. The decision of how to restore the resulting facial defect, either surgically or prosthetically, is often determined by a combination of patient and physician factors.

Surgical reconstruction may not be a simple task depending upon the size of the defect and may require multiple surgeries to obtain an acceptable result. Total treatment time may vary; however, completion of surgical nasal reconstruction has been reported to be upward of 26 months.^2^ Additionally, surgical reconstruction may have esthetic limitations in the ability to match the color and contour of the reconstructed nose compared to the preoperative appearance. For elderly or medically compromised patients that are poor candidates for prolonged treatment courses that are likely to require multiple surgeries, alternative treatment options should be considered.

As an alternative, reconstruction of a nasal defect with a prosthesis is expeditious and can faithfully replicate the missing facial structure.^3^ Conventional nasal prosthesis fabrication can be completed when the surgical bed is well‐healed, and the completed prosthesis is retained by medical grade adhesive. The interval for adhesive reapplication on the prosthesis is patient‐specific. Additionally, patients are required to clean the adhesive from their prosthesis and their skin on a daily basis. This may be challenging for a geriatric or nondexterous patient, and the patient may require additional assistance. To alleviate these challenges, craniofacial implants can be utilized to retain the nasal prosthesis.

Craniofacial implants, often fabricated in titanium, are a reliable technique for maxillofacial reconstruction with reported implant success rates of 70%‐80%.^4^, ^5^, ^6^ The location of the craniofacial implant has been reported to impact success with nasal implant success ranging from 71.4% to 100%,^2^, ^7^, ^8^ and orbital implant success ranging from 27% (irradiated implant sites) to 75% (nonradiated implant sites).^2^, ^8^, ^9^ Dental implants have been reported to be successful in geriatric patients^10^ but there are limited reports on the role of craniofacial implants in a geriatric oncologic population. The purpose of the study is to describe the process for management of a geriatric patient with squamous cell carcinoma of the nose reconstructed with a treatment workflow utilizing craniofacial implants.

## 2 CASE REPORT

**Figure 1:**
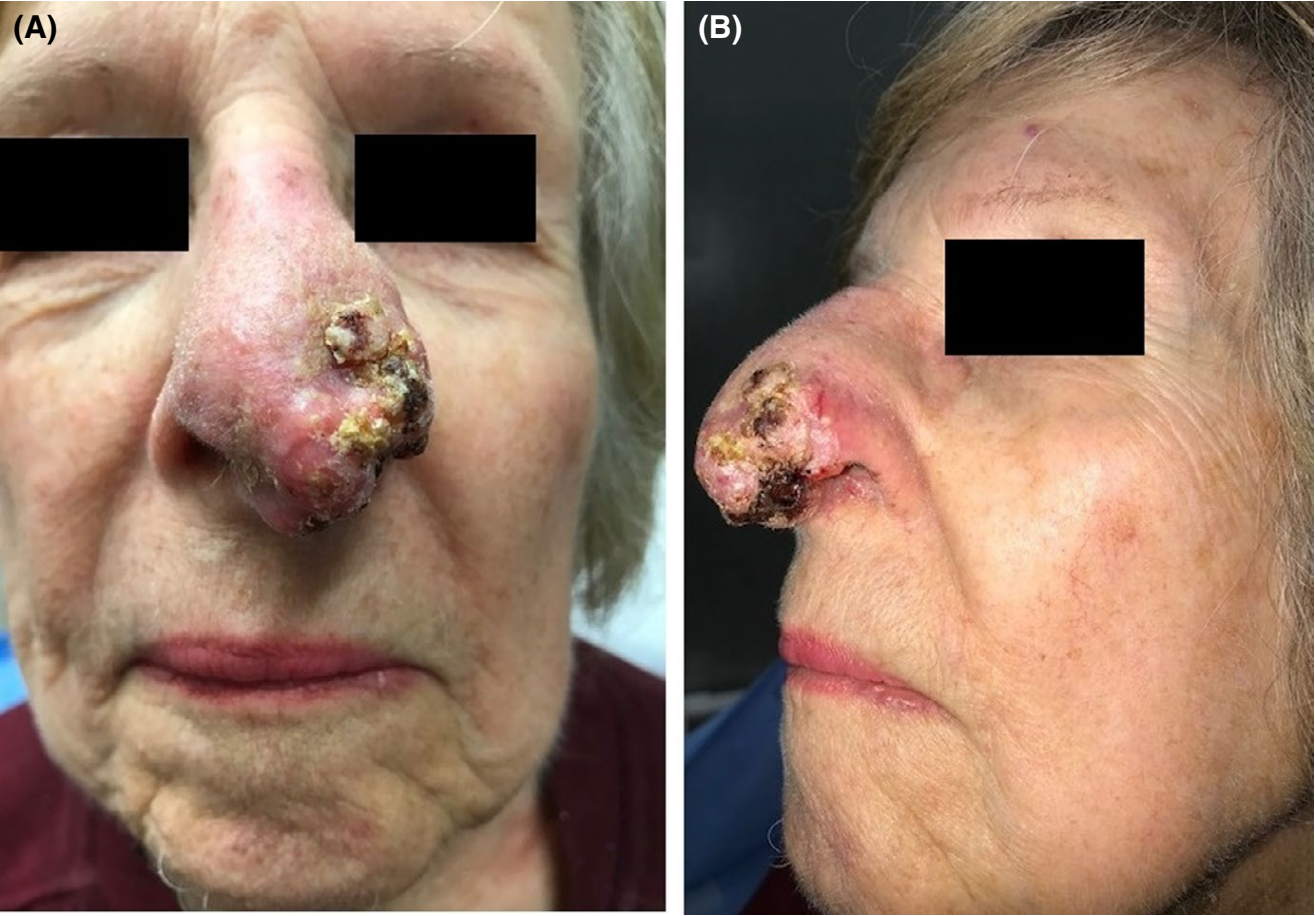
A, Frontal view of preoperative view of the squamous cell carcinoma of the nose. B, Lateral view of preoperative view of the squamous cell carcinoma of the nose

An 86‐year‐old female patient presented to the Head and Neck Service at Memorial Sloan Kettering Cancer Center for management of a locally advanced squamous cell carcinoma of the left nose (Figure 1A,B). The patient was recommended to have a total rhinectomy with bilateral modified neck dissection with reconstruction of the nasal defect with a nasal prosthesis. The oncological resection was carried out by the head and neck surgical team. Subsequent pathology showed an advanced primary cancer with clear surgical margins and with no pathological involved lymph nodes. As such, no postoperative radiation was recommended. After a discussion of the risks and benefits of treatment, including the course of treatment for surgical and/or prosthetic reconstruction, the patient elected to have her planned nasal defect prosthetically reconstructed. The patient was preoperatively evaluated by the Dental Service, and a nasal moulage was made. The patient was then planned for craniofacial implants to facilitate retention of the nasal prosthesis.

Total rhinectomy with bilateral modified neck dissection was performed by the head and neck team. A provisional nasal prosthesis^11^ was delivered postoperatively which replicated the patient's nasal contours. The prosthesis was secured with three pieces of medical grade adhesive tape and was removed daily by the patient.

**Figure 2:**
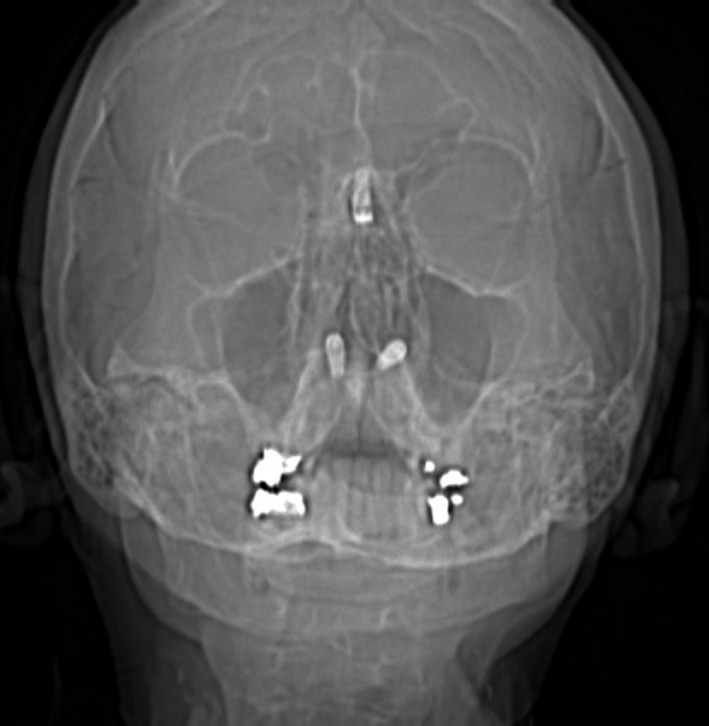
CT showing the three implants placed for prosthetic replacement support

A postoperative CBCT was completed for craniofacial implant planning. A nasal surgical stent was fabricated to assist in the accurate placement of the craniofacial implants based on the surgical plan. The patient was then brought back to the operating room 2 weeks following rhinectomy for placement of the craniofacial implants. The surgical stent was utilized to identify the planned implant locations intraoperatively. An incision was made on the nasal floor exposing the premaxilla and vertically along the glabellar skin to expose the bone in the glabellar region. Three osteotomies were created, two in the premaxilla region and one in the glabellar region, and three 4 mm craniofacial implants (Vistafix VXI300; Cochlear) were then placed to the proper depth with adequate primary stability. Three sterile cover screws were placed on the implants and were hand‐tightened (Figure 2). Primary closure was achieved with 3‐0 vicryl sutures on the nasal floor and 5‐0 nylon sutures in the glabellar region. Xeroform packing was placed within the nasal cavity. The patient was counseled to return for follow‐up in 2‐3 months to begin fabrication of a definitive nasal prosthesis.

The patient returned to the Dental Service 3 months status postsurgery for uncovery of the craniofacial implants. Topical betadine was administered to the skin surrounding the nasal defect, and local anesthesia (68 mg 2% lidocaine with 1:100 000 epinephrine) was directly administered to the skin surrounding the implant sites. The implant in the glabellar region did not require tissue excision as the implant platform was exposed. The cover screw of the superior implant was removed, and a 7.5 mm sterile healing abutment was placed and hand‐tightened. Then, two soft tissue punches were completed using a 4 mm‐tissue punch, exposing the two implants in the premaxilla area and two sterile 7.5 mm healing abutments were placed and hand‐tightened. Xeroform gauze was used around the abutments in the premaxilla to compress the adjacent skin, and the patient was counseled to use bacitracin for postoperative wound care.

**Figure 3:**
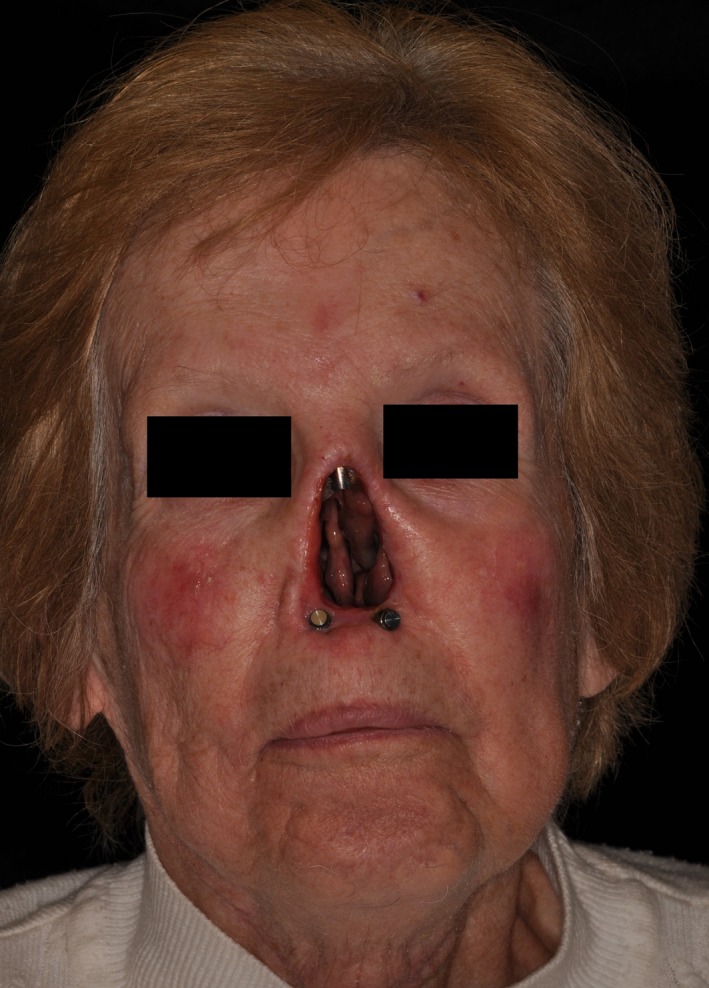
Frontal view showing postoperative complete healing postimplant placement

After 1 month, the patient returned to begin fabrication of the nasal prosthesis. A nasal moulage was completed using irreversible hydrocolloid impression material (Jeltrate Plus; Dentsply Sirona) and fast setting plaster (Type V, Diekeen green; Kulzer Dental). Then, the healing abutments were removed and replaced with 7.5 mm final abutments (Vistafix VXA300; Cochlear) and were torqued to 25 Ncm. Three prosthesis magnets were placed: one on the abutment in the glabellar region (Maxilip magnet; Factor II Inc) and 2 on the abutments in the premaxilla region (Minilip magnet; Factor II Inc) (Figure 3). Magnetic impression copings (S‐range, Factor II Inc) that had been luted together with acrylic resin (Jet, Lang Dental Manufacturing Co., Inc) were used to complete another facial moulage with irreversible hydrocolloid (Jeltrate Plus; Dentsply Sirona) and fast setting plaster (Type V, Diekeen green; Kulzer Dental) to transfer the implant locations onto a model of the patient.

**Figure 4:**
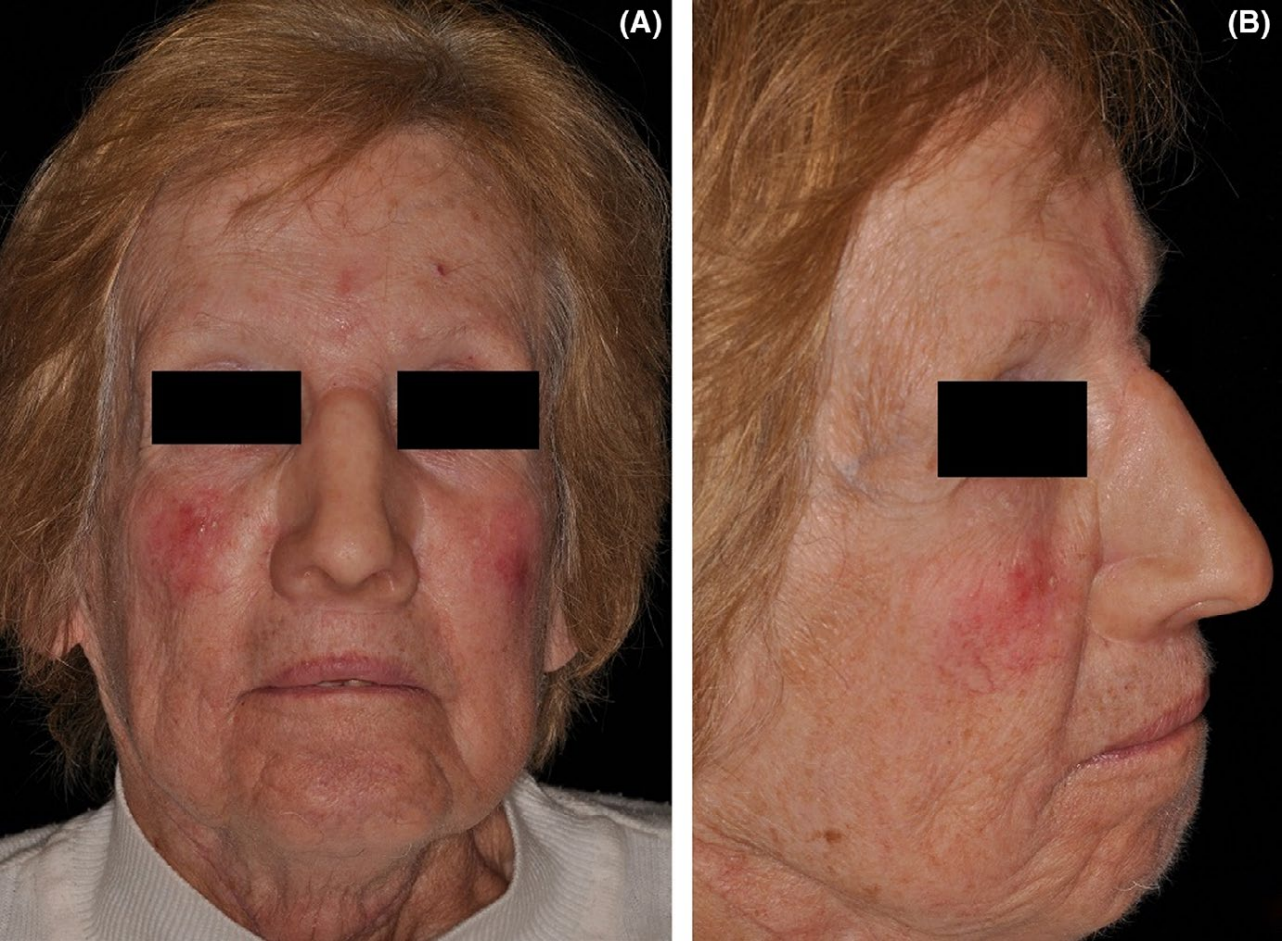
A, Frontal postoperative view with the nasal prosthesis in place. B, Lateral postoperative view with the nasal prosthesis in place

Using the patient's model, a wax sculpture of the nasal prosthesis was then made and was subsequently tried on the patient. A custom magnetic keeper (S‐range, Factor II Inc) was tried on the magnetic abutments and was properly adapted. A base shade for the nasal prosthesis was selected followed by processing of the prosthesis into silicone (RTV 40, Factor II Inc) which was extrinsically tinted to match the patient's adjacent skin colors. The completed nasal prosthesis was then delivered (total treatment time 8 months following surgery) (Figure 4A,B), and home care instructions were reviewed. The patient and family were very satisfied with the esthetics, fit, and function of the nasal prosthesis.

## 3 DISCUSSION

Acquired nasal defects are debilitating deformities that require preplanning for adequate reconstruction. For the geriatric patient, the use of adjuvant therapy, the risk of disease recurrence, systemic comorbidities, and known complications of general anesthesia may diminish enthusiasm from a conventional approach for surgical reconstruction. Moreover, a reconstructive plan requiring multiple surgeries and prolonged treatment time may be undesirable for this patient population. To optimize outcomes, inclusion of supportive care team members (ie, including nursing, counseling, and social work) has previously been previously reported.^12^, ^13^, ^14^

Prosthetic rehabilitation of a nasal defect is an alternative option for geriatric patients which does not require additional surgery, is safe, and has a shorter treatment time. Additionally, the use of craniofacial implants is desirable for this patient population to assist in prosthesis retention. As in this report, a magnet retention system may assist a geriatric patient in positioning the prosthesis during prosthesis placement. The use of a medical grade adhesive can be completely avoided which may be desirable for a nondexterous patient. Home hygiene of the implant abutments is required; however, localized implant dermatitis/mucositis (3%‐60% depending on the severity of the reaction)^7^, ^15^ is the most commonly described biologic complication. Management is usually limited to local control with tissue excision.^3^

If the outlined approach is being considered for a geriatric patient undergoing oncologic resection, primary placement of craniofacial implants during the primary oncologic surgery is desirable. This eliminates the need for additional surgeries for implant placement as well as minimizes overall treatment time. Moreover, a multidisciplinary treatment team inclusive of supportive and rehabilitation medicine specialists is a prerequisite for successful execution of oncologic resection, implant planning and placement, and prosthetic fabrication.

## 4 CONCLUSION

For an oncologic aging patient unable or unwilling to undergo surgical reconstruction of a nasal defect posttotal rhinectomy, prosthetic rehabilitation with craniofacial implants offers an expeditious reconstructive approach. Multidisciplinary care is needed for a satisfactory outcome.

## CONFLICT OF INTEREST

None declared.

## AUTHOR’S CONTRIBUTION

All authors, EBR, ZUA, JMH, IG are equally contributed to the design, analysis, and presentation of this study. EBR: involved in study design, writing, and revision. ZUA: involved in study design, writing, and revision. JMH: involved in study design and final revision. IG: involved in study design and final revision.
